# Biofortification: an approach to eradicate micronutrient deficiency

**DOI:** 10.3389/fnut.2023.1233070

**Published:** 2023-09-14

**Authors:** Avnee, Sonia Sood, Desh Raj Chaudhary, Pooja Jhorar, Ranbir Singh Rana

**Affiliations:** ^1^Department of Agronomy, CSK Himachal Pradesh Krishi Vishvavidyalaya, Palampur, India; ^2^Department of Vegetable Science and Floriculture, CSK Himachal Pradesh Krishi Vishvavidyalaya, Palampur, India

**Keywords:** biofortification, hidden hunger, selective breeding, sustainable, vitamins and minerals

## Abstract

Micronutrient deficiency also known as “hidden hunger” refers to a condition that occurs when the body lacks essential vitamins and minerals that are required in small amounts for proper growth, development and overall health. These deficiencies are particularly common in developing countries, where a lack of access to a varied and nutritious diet makes it difficult for people to get the micronutrients they need. Micronutrient supplementation has been a topic of interest, especially during the Covid-19 pandemic, due to its potential role in supporting immune function and overall health. Iron (Fe), zinc (Zn), iodine (I), and selenium (Se) deficiency in humans are significant food-related issues worldwide. Biofortification is a sustainable strategy that has been developed to address micronutrient deficiencies by increasing the levels of essential vitamins and minerals in staple crops that are widely consumed by people in affected communities. There are a number of agricultural techniques for biofortification, including selective breeding of crops to have higher levels of specific nutrients, agronomic approach using fertilizers and other inputs to increase nutrient uptake by crops and transgenic approach. The agronomic approach offers a temporary but speedy solution while the genetic approach (breeding and transgenic) is the long-term solution but requires time to develop a nutrient-rich variety.

## Introduction

Over 2 billion people worldwide suffer from micronutrient deficiency which has a negative impact on their health and socio-economic condition (1). The principal reason is the consumption of cereal-based food which provide enough calories but they are deficient in phytochemicals (minerals, vitamins, antioxidants, and fiber). These phytochemicals are essential for the normal growth and development of humans and their deficiencies can have serious health consequences, including diminished cognitive degeneration in children, increased risk of infections and a range of other negative effects on physical and mental health.

Green Revolution, which took place from the mid-20th century onwards, marked a significant shift in agricultural practices and policies, particularly in developing countries like India. During the revolution, the emphasis was shifted on boosting crop productivity, notably that of rice and wheat, which led to the domination of these two crops in the nation. The increased productivity ensured food security in the country but on the other hand, decreased bio-diversity resulted in a monotonous cereal-based diet and thus increased concern about nutritional security. The ever-growing population of India further worsens the problem of providing sufficient nutrients to all. Over 21.9% of the Indian population is living in extreme poverty with limited access to resources (2). With their poor purchasing power, they consume what they produce in their fields. In order to alleviate malnutrition and to attain nutritional security in the country, a second green revolution is therefore required, with a particular emphasis on the development of biofortified, nutrient-rich varieties.

**Figure 1:**
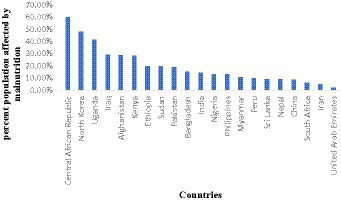
Different countries and their percentage of population suffering from malnutrition.

Staple crops, such as rice, wheat and maize are the main source of calories for a large proportion of the world’s population, particularly in low-income countries. These crops, however, often lack essential vitamins and minerals, which might result in micronutrient deficiency. Biofortification can help to address this problem more sustainably and economically by increasing the levels of essential vitamins and minerals in staple crops. It is the process of enrichment of bio-available concentration in edible portions of food and aims at providing nutrient-rich food to rural resource-poor people who do not have access to diversified food, supplements and industrially fortified food (see Figure 1). Biofortification can be a cost-effective and sustainable way to address micronutrient malnutrition at the population level with an ultimate goal to reduce malnutrition and improve public health, particularly in populations that rely heavily on a single staple crop for their daily caloric intake. It requires a one-time investment unlike supplements, reach malnourished poor population and provide better quality food without compromising yield (see Figure 2). This can be particularly important in developing countries where micronutrient deficiencies are common and can have serious health consequences. Iron, zinc, iodine and selenium deficiencies are the most common, which account for around 60% of iron, 30% of zinc and iodine and 15% of selenium deficiency (3). However, biofortification alone is not enough to eradicate malnutrition. It cannot provide such a high level of nutrients as through supplements or fortified food but they improve daily dietary intake of nutrients (4). In the context of climate change also, the anticipated drop in dietary micronutrients makes biofortification more important for vulnerable groups to maintain good health.

The concept of simultaneously biofortifying crops with multiple essential micronutrients is an innovative and promising strategy to address widespread nutrient deficiencies and improve overall human nutrition. This approach, often referred to as “multi-nutrient biofortification” or “combinatorial biofortification,” aims to create crops that contain a balanced array of various essential vitamins and minerals. This strategy can provide a more holistic and comprehensive approach to combating malnutrition by addressing multiple nutrient deficiencies concurrently. By combining different nutrients in crops, their overall bioavailability and health benefits can be maximized. Multi-nutrient biofortified crops can encourage dietary diversity as people consume a wider range of nutrients from staple foods. In addition to cost savings from development to distribution and synergies through aggregated health effects, multi-nutrient biofortification can result in significantly higher market coverage by preventing competition between numerous single-nutrient biofortified varieties (5).

## Approaches of biofortification

**Figure 2:**
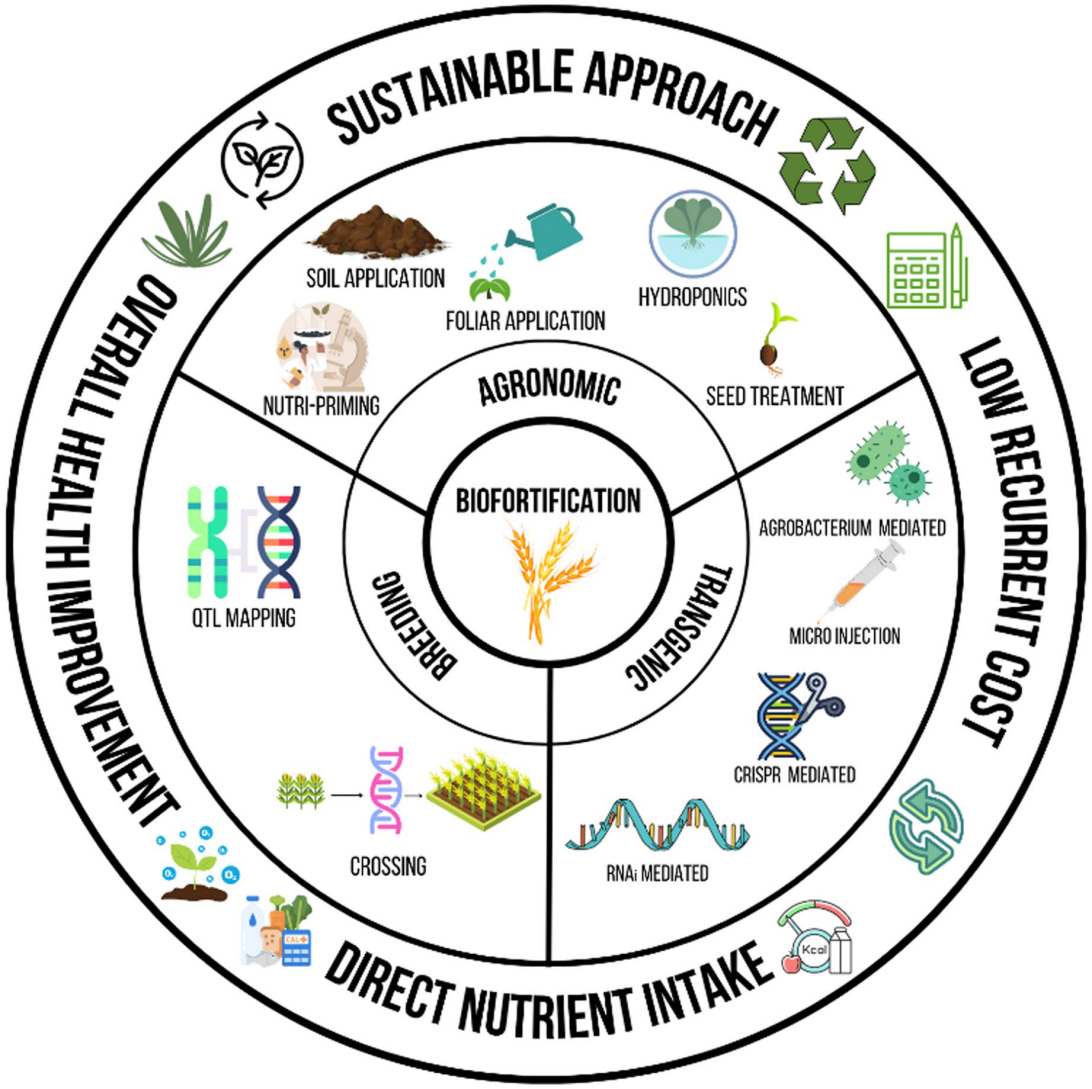
Methods of biofortification (Agronomic, Breeding and Transgenic).

## Agronomic biofortification

Agronomic biofortification refers to the process of enriching the nutritional value of crops through fertilization and soil management. It offers an efficient and timely solution that is the quickest and most affordable way to produce nutrient-dense food, albeit it only offers a short-term fix. The majority of crops can benefit from this very simple method of biofortifying with iron, zinc, iodine, and selenium. To boost the content of micronutrients in the plant’s edible parts, micronutrient-containing organic/inorganic fertilizers or biofertilizers are applied to the plant by foliar or soil application. Micronutrient concentration depends on the source of fertilizer used, method and rate of fertilizer application, stage of application, and translocation of nutrients within plants. Due to variations in mineral mobility, mineral accumulation among plant species and soil compositions in the particular geographic region of each crop, the success of agronomical biofortification is highly variable (6). The effectiveness of agronomic biofortification has increased with the development of high specialized fertilizers with high nutrient uptake efficiency and greater nutrient translocation to the consumable sections of a crop plant, which include water-soluble fertilizers, chelated fertilizers and nano-fertilizers (7).

Nanoparticles, which are extremely small particles with unique properties due to their size and structure, have gained attention as potential tools for biofortification. Nanofertilizers (NFs), a subset of nanotechnology applications in agriculture, hold significant potential to revolutionize traditional methods of enhancing crop nutrient content and improving overall nutritional quality. By harnessing the unique properties of nanoparticles, NFs offer a promising avenue for increasing the concentration and bioavailability of essential nutrients in food crops, a strategy known as “nanofertilizer-assisted biofortification.” Because of the large increase in surface area and the NFs’ small size, plants can easily absorb the particles (8).

### Iron biofortification

Iron deficiency is a common problem, particularly in developing countries and it can have serious health consequences such as anemia affecting over half the population of children under the age of five and pregnant women in India. Biofortification can help to address this issue by increasing the iron content of crops, which can in turn help to improve the iron intake of individuals who rely on these crops as a dietary staple. Iron is a vital nutrient for humans, it is required for proper body functioning but cannot be produced by the body and must be obtained from the diet. It is crucial for the production of red blood cells and the transportation of oxygen within the human body, supporting the immune system, providing energy and maintaining healthy skin, hair and nails. It is especially important for pregnant women, infants, and young children, as they have higher iron requirements. Anemia, weariness, and immune system impairment are just a few of the health issues that iron deficiency can cause, especially in impoverished nations where plant-based foods are the main source of Fe (9).

Applying iron-rich fertilizers to the soil and proper soil management, such as maintaining the pH and nutrient balance of the soil can help to improve the uptake of iron by crops. It is important to note that the efficacy of these methods can vary based on the particular crop and growing circumstances. Comparing vegans to meat-eaters, the former should consume 1.8 times more of the recommended daily intake (RDA) of Iron than later (10). Table 1 shows some of the crops that are successfully agronomically biofortified with iron.

**Table 1:** Agronomic biofortification of iron.

Crop	Treatment	References
*Cereals*		
Rice	Foliar application of FeSO₄ sprayed @ 0.2% at panicle initiation stage, 7 days after flowering (DAF), 14 DAF	(11)
Brown rice	0.5 and 1.0% FeSO_4_. 7H_2_O at maximum tillering, pre-anthesis and post-anthesis stages	(12)
Aerobic rice	Soil application of 67 mg FeSO₄.7H₂O per kg soil at the time of sowing; three foliar sprays (at 40, 60 and 75 DAS) of 3% FeSO₄.7H₂O solution and 0.05 M Fe-EDTA used as seed treatment	(13)
Wheat	Three foliar sprays of FeSO₄ at tillering, booting and heading stage	(14)
	Foliar application of Fe_3_O_4_ nanofertilizer (5 mg L^−1^)	(15)
*Pulses*		
Mungbean	Foliar application of 0.5, 1 and 1.5% solutions of FeSO_4_ at branching and flowering stages. Increased Fe concentration by 46% in grains.	(16)
Cowpea	Four foliar application rates (0, 25, 50 and 100 μM L^−1^) each of iron chelates and ferrous sulfate. Increased Fe concentration by 29–32%.	(17)
Chickpea	Foliar application of FeSO_4_.7H2O resulting in an increased grain concentration by 21–22%	(18)
Lentil	Foliar spray of 0.5% FeSO_4_.7H2O at the pre-flowering stage. Increase in concentration by 17.4 mg kg^−1^ in grains.	(19)
*Vegetables*		
Potato	Soil (Amino acid-based Fe complex) and foliar applied EDTA chelated Fe	(20)
Red and green pigmented Lettuce	Soilless culture: Fe at conc. 0.5, 1.0 and 2.0 mM iron	(21)
Brassicaceae microgreens (Arugula, red cabbage, and red mustard)	Soilless media: Fe conc. 0, 10, 20, 40 mg L^−1^	(22)

### Zinc biofortification

Zinc is a crucial micronutrient that is necessary for both plants and humans. It is involved in a wide range of physiological functions including immune response, protein and DNA synthesis, wound healing and involved in the metabolism of carbohydrates, proteins and fats. Zinc deficiency is a major public health problem affecting around 30% of the world’s population, making people more susceptible to issues including maternal mortality, DNA damage, growth retardation, changes in taste and smell, immunological dysfunction, and an increased risk of infections (23).

Biofortification can assist to solve the issue of zinc deficiency and improve the nutritional value of crops, particularly in areas where deficiency is prevalent. Zinc deficiency in humans and soil show geographical overlap (24). A high percentage of agricultural land (36.5%) in India is zinc deficient and cultivating crops on these deficient soils further reduces zinc levels in edible portions (25). The deficit is particularly prevalent in low-income developing nations where a plant-based diet is the norm (26). Agronomic biofortification of zinc was reported to be successful in a large number of crops (Table 2).

**Table 2:** Agronomic biofortification of zinc.

Crop	Treatment	References
*Cereals*		
Maize	Soil application of ZnSO₄.7H₂O @ 2.5, 5 and 7.5 kg/ha	(27)
Aromatic rice	Soil and foliar application (at flowering) of zinc as ZnSO₄.7H₂O	(28)
Basmati rice	Different Zn sources applied at the rate 5 kg Zn/ha	Singh and Shivay (29)
Rice	Soil and foliar sprays (two) at maximum tillering and panicle initiation stage with different sources of zinc at different rates	(30)
	Soil and foliar application of ZnSO₄.7H₂O and zinc-coated urea	Prasad and Shivay (31)
Wheat	Soil and foliar sprays (two) at maximum tillering and booting stage with different sources of zinc at different rates	(32)
	Soil and foliar application of zinc as ZnSO₄.H₂O	(33)
	Dextran sulfate (DEX (SO4))-coated ZnO Nanoparticles	Elhaj and Unrine (34)
	Different rates and source of Zinc at 1/3- sowing, 1/3–3 weeks after sowing (WAS) and 1/3–6 WAS	Prasad and Shivay (31)
Bread wheat, durum wheat and triticale	Foliar application of 0.5% ZnSO₄.7H₂O at maximum tillering, flower initiation, milk and dough stages. The maximum percent increase was 145.9% in wheat, 178.1% in durum wheat and 157.4% in triticale	(35)
*Pulses*		
Chickpea	Soil application (5 kg/ha) through ZnSO_4_.7H2O Increase in concentration of zinc by 5.4 mg kg^−1^	(36)
Pigeon pea	Soil application (20 kg/ha) ZnSO_4_.7H2O Increase in concentration of zinc by 10.6 mg kg^−1^	Behera et al. (37)
Lentil	Foliar spray of 0.5% ZnSO_4_.7H2O at the pre-flowering stage. Increase in concentration by 10.5 mg kg^−1^ in grains.	(19)
*Oilseeds*		
Safflower	Soil application (5 kg/ha) through ZnSO_4_.7H2O	Roy and Ghosh (38)
Linseed	Foliar application of ZnSO_4_.7H_2_O and Zn-EDTA (0, 0.25, 0.50, and 0.75%) at flowering and capsule formation stages	(39)
*Vegetables*		
Brassicaceae microgreens (Arugula, red cabbage, and red mustard)	Soilless media: ZnSO_4_ (5–10 mg/L) 75–281% increase in zinc content in crop	(22)
Arugula (Broad leaf)	Foliar application (Zn @ 1.5 kg/ha at 25 DAE) 279% increase in Zn content	(40)
Lettuce	Different Zn doses (0,5, 10, 20, 30 mg kg^−1^)	(41)
Broccoli	0.25% ZnSO_4._ 7 H_2_O @ 15 mL per pot	(42)
*Fruits*		
Pear	Foliar spray of 1.5% ZnEDTA	(43)
Banana	Injection of ZnSO_4._ 7 H_2_O in pseudostem @ 1, 2 and 4% Triple zinc content in fruit than control	(44)

### Iodine biofortification

Iodine is a necessary component of human metabolism and crucial for the proper function of the thyroid gland in humans, energy production and body temperature regulation, despite not being a necessary element for plants, although its application has been linked to higher yields and high iodine content in several crops (Table 3). It is estimated that the iodine intake of 30–38% of people worldwide is insufficient (45).

**Table 3:** Agronomic biofortification of iodine.

Crop	Treatment	References
Tomato	Potassium iodide (KI), KIO_3_ + SA, KI + SA @ I-7.88 μM and 7.24 μM	(45)
Pepper	KI at 0.25 to 1 mg/L Increased iodine content by 350–1,330 μg/kg	(46)
Cabbage and cowpea	Potassium iodide @ 15 kg I /ha Increase in iodine conc. to 109.1 mg/kg in cabbage and 5854.2 mg/kg in cowpea	(47)
Carrot	Iodine dose at 0.5 mg/L	(48)
Potato	KIO_3_ @ 2.0 kg iodine per hectare	(49)
Apple and Pear	Foliar application of KI and KIO_3_ (0.5, 1.0 and 2.5%)	(50)
Leafy greens (Rapeseed and Amaranthus)	Soil and foliar Iodine application at different concentrations (0, 5, 10 kg/ha)	(51)
Sweet basil and lettuce	KI @10 μM	(52)

Iodine deficiency can lead to a variety of health problems, including goiter, infertility, growth impairment, hypothyroidism and intellectual disability. Iodine biofortification can be a particularly useful approach in areas where iodine deficiency is common and people rely heavily on plant-based foods as a source of nutrients. Biofortified plant-based foods can help to increase the overall iodine intake of a population and improve the nutritional status of individuals who consume these foods. Adults should consume between 150 to 290 μg of iodine per day, with a tolerable upper limit of 1,100 μg per day (53, 54). One of the most common ways to fortify iodine is through the addition of iodine to salt. This is called iodized salt. Iodized salt may not be effective in preventing iodine deficiency in all populations as it can raise blood pressure which is a major risk factor for heart disease and stroke. It is also linked to an increase in cases of osteoporosis, a condition that causes the bones to become weak and brittle. A promising strategy to raise the iodine content of crops is agronomic biofortification.

### Selenium biofortification

Selenium is a trace element that is essential for human health. Due to its incorporation into selenoproteins like glutathione peroxidase, which perform a number of functions including antioxidant activity, it is essential for the immune system’s proper operation (55). Se deficiency affects hundreds of millions of people around the world (56). For plants, Se is not essential (57, 58), but when applied at low doses, it is beneficial for some groups of plants by increasing the activity of various enzyme systems; selenium alone or in combination with iodine was found to increase concentration, better quality in some plants (Table 4); for example, it delays tomato fruit ripening by inhibiting ethylene biosynthesis and enhancing the antioxidant defense system (80).

**Table 4:** Agronomic biofortification of selenium.

Crop	Targeted micro-nutrient	Treatment	References
*Pulses*			
Chickpea	Selenium	Two foliar Se fertilizers (sodium selenate and sodium selenite) at four rates (0, 10, 20, 40 g ha^−1^)	(59)
Soybean	Selenium	Foliar application of sodium selenite and Se-enriched fertilizer	(60)
Lentil	Selenium	Foliar application of 40 g/ha of Se as potassium selenate (K_2_SeO_4_) (10 g/ha during full bloom and 30 g/ha during the flat pod stage) Increased seed Se concentration from 201 to 2,772 *μ*g/kg	(61)
*Oilseeds*			
Mustard	Selenium	Accumulation of 358 mg kg^−1^ in seed	(62)
*Root crops*			
Carrot and Broccoli	Selenium	Selenium conc. at 1.65 mg/kg, 0.92 mg/kg and 88 μg/L, 48.6 μg/L	(63)
Carrot	Iodine and selenium	KI + Na_2_SeO_3_ & KIO_3_ + Na_2_SeO_3_	(64)
Turnip	Selenium	Selenite at 50 to 100 mg L^−1^	(65)
Radish	Selenium	Selenate and selenite @ 20 μmol L^−1^	(66)
Carrot	Iodine and selenium	KI + Na_2_SeO_4_ (4 kg I /ha: 0.25 kg /ha) Iodine and selenium content (7.7 and 4.9 times)	(67)
Carrot	Iodine and selenium	KI and Na_2_ SeO_4_ (4 kg I ha^−1^: 0.25 kg Se ha^−1^)	(68)
*Cole crops*			
Broccoli	Selenium	Se dose, selenite and selenate	(69)
Cabbage	Selenium	Na_2_O_4_Se: betaine: adjuvant (10 μM: 10 μM: 1%)	(70)
Broccoli and Carrot	Selenium	Selenium enriched *S. pinnata* (soil amendment)	(71)
Broccoli	Selenium	Sodium selenate (50 μM)	(72)
Mustard sprout	Selenium	Selenate and selenite @ 20 μmol L^−1^	(66)
Cabbage	Selenium	8 mg kg^−1^ and 16 mg kg^−1^ Se yeast	(73)
*Cucurbits crops*			
Pumpkin	Selenium and iodine	Selenium and iodine combination	(74)
*Leafy vegetables*			
Lettuce	Selenium	Selenate at 40 μmol L^−1^	(75)
Lettuce	Selenium	Selenate application at low concentration	(58)
Lettuce	Iodine and selenium	Foliar application of Na_2_SeO_4_ and Na_2_SeO_4_ + KIO_3_ and for iodine content in roots KIO_3_ and Na_2_SeO_4_ + KIO_3_	(76)
*Solanaceous vegetables*			
Tomato	Selenium	Selenium dose at 10 mg L^−1^	(77)
Tomato	Selenium	Sodium selenite at 5 mg L^−1^ Accelerated accumulation of selenium by 53%	(78)
Cherry tomato	Selenium	Selenium at 2.0–4.0 μmol L^−1^ + grafting (soilless media) Se concentration (9.8 mg kg^−1^)	(79)

Plants can absorb selenium from the soil, but the availability of selenium in the soil can vary widely. In some areas, the soil may be naturally low in selenium, while in others, selenium may be present but not in a form that plants can easily absorb. To ensure that plants are getting sufficient selenium, it may be necessary to add selenium to the soil. This can be done through the use of selenium fertilizers or through the application of selenium-rich compost or manure. It is also possible to provide selenium to plants through the use of selenium-rich irrigation water or through the use of selenium-enriched seeds.

### Interaction among nutrients

The effectiveness of agronomic biofortification can be affected by interactions between macronutrients and micronutrients (81) (see Table 5). While previous biofortification initiatives have mainly concentrated on increasing specific nutrients, a novel strategy could be to simultaneously biofortify crops with a number of essential micronutrients, providing a more comprehensive nutritional profile. Some nutrients work synergistically in the body, enhancing the absorption and utilization of others. Zinc, for example, improves nitrogen metabolism by promoting effective uptake and assimilation. It also aids in the conversion of phosphorus into forms that are easily absorbed by plants, as well as in the regulation of stomatal function and water movement, both of which affect potassium absorption. The synthesis of selenium-containing phytochemicals, such as selenocompounds can be improved by selenium biofortification. According to (82, 83), plants convert selenium into selenoamino acids, which are then converted into phytochemicals. These substances have antioxidant qualities beneficial to human health. Selenium also plays a role in reducing element toxicity and regulating the concentration of micronutrients in plants by modifying soil conditions, encouraging microbial activity, taking part in crucial physiological and metabolic processes, generating element competition, stimulating metal chelation, organelle compartmentalization and sequestration (84). It is preferable to combine Zn, Se, and Fe in conjunction with the employment of plant growth-promoting bacteria (PGPR) and arbuscular mycorrhizal fungus (AMF), in order to develop biofertilizers that are ecologically benign yet result in crops that are enriched in microelements (85).

**Table 5:** Effect of nutrients on other nutrients and phytochemicals.

Crop	Targeted nutrient	Treatment	Improved traits	References
*Legume crops*				
Green beans	Macro (N, P, K, Ca, and Mg) and Micronutrients (Fe, Mn, Zn, Cu, and Ni)	Zn Chelate and Zn Sulphate (25 to 50 μM)	Antioxidant activity and macro and micro-nutrient content	(90)
*Oilseeds*				
Mustard	Boron and nitrogen	Borax (0.5 and 1%) and urea (1%)	Increased oil and protein content	(91)
*Cole crops*				
Cabbage	Selenium	8 mg kg^−1^ and 16 mg kg^−1^ Se yeast	Antioxidant activity and nutritional quality (ascorbic acid, soluble sugar, free amino acids, SOD activity, Glucosinolates, and Phenolic compounds)	(73)
Broccoli	Nitrogen and Zinc	0.25 + 0.25 of N and Zn (foliar application)	Antioxidant activity, Zn (more than 50 mg K^−1^) and total phenol content	(92)
*Cucurbits crops*				
Cucumber	Potassium	Potassium sulphate at 0.014–4 g L^−1^	Potassium content	(93)
*Leafy vegetables*				
Spinach	PSB	PSB + FYM applied at different stages	Micronutrients (Ca, Mg, Zn and Fe), vitamin C and beta-carotene	(94)
Lamb’s Lettuce	Selenium	Soil and foliar application of Selenium	Phenolic compounds	(95)
Alfalfa	Selenium	Selenate and selenite @ 20 μmol L^−1^	Anthocyanin concentration	(66)
*Solanaceous vegetables*				
Pepper	Iodine	Hydroponic experiment: KI conc. 0.25–5 mg L^−1^	Ascorbic acid	(46)
Tomato	Selenium	Selenium dose at 10 mg L^−1^	Ascorbic acid, soluble sugar, chlorophyll-a content, peroxidase, catalase, and superoxidase dismutase	(67)
Tomato	Selenium	Sodium selenite at 2 mg L^−1^	Biosynthesis of phytochemical compound	(96)
*Root vegetable*				
Turnip	Selenium	Selenite at 50 to 100 mg L^−1^	Selenium content and other minerals *viz.*, Mg, P, Zn, Mn, and Cu.	(65)

In some cases, certain nutrients can compete with each other for absorption. By ensuring an adequate balance of multiple nutrients, competition for absorption can be minimized. Adequate levels of nutrients like magnesium, zinc and manganese are important for vitamin C synthesis. These nutrients can impact the enzyme systems involved in ascorbic acid production. Certain micronutrients, such as copper and manganese, are cofactors for enzymes involved in the synthesis of antioxidants and flavonoids (86). Adequate levels of these micronutrients can positively influence the production of these compounds. The formation of roots, the movement of shoots and the re-localization of nutrients from vegetative tissue to the seeds are all positively impacted by a plant’s N and P status. As a result, the crop’s edible sections absorb more micronutrients and have higher concentrations of them (87).

Salt, high/low temperature, heavy metals, and drought all cause the overproduction of reactive oxygen species (ROS) and the induction of oxidative stress in plants (85). It has been demonstrated that biofortification with Zn, Se, and Fe using various types and forms of fertilizer can reduce the damage caused by oxidative stress by increasing the content of ROS-scavenging enzymes such as superoxide dismutase (SOD), ascorbate peroxidase (APX), catalase (CAT), glutathione peroxidase (GPX), monodehydroascorbate reductase (MDHAR), dehydroascorbate reductase (DHAR), glutathione reductase (GR), glutathione S-transferase (GST), and peroxiredoxin (PRX) content in different sites of plant cells (88, 89).

## Biofortification through conventional breeding

Traditional plant breeding is a method of improving the nutritional content of crops by selecting for desired traits through controlled crosses between different plant varieties. The process involves selecting plants with desirable traits, such as higher micronutrient content and crossing them with other plants to create a hybrid with improved characteristics. Over time, this process is repeated and the offspring are screened for desired traits.

Plant breeding techniques are used in the biofortification approach to develop staple food crops with greater micronutrient content (97), this assists to target low-income households in the country. Numerous crops have been targeted for biofortification through crop breeding due to their improved acceptance (Table 6). A biofortified crop system is highly sustainable. Nutritionally improved varieties will continue to be grown and consumed year after year, even if government attention and international funding for micronutrient issues fade (105). Since the last four decades, yield qualities and resistance breeding have received the majority of attention resulting in lower amounts of nutrients in the existing varieties (106).

**Table 6:** Varieties developed through conventional breeding.

Crop	Variety	Trait	Country	Year release	References
*Cereals*					
Rice	DRR Dhan 45	Zn	India	2016	(98)
	DRR Dhan 48, DRR Dhan 49	Zn	India	2017	(98)
	Saurbhi	Zn	India	2017	(98)
	Fedearroz BIOZn 035	Zn	Colombia	2021	(99)
	Inpara 11 Siam HiZInc	Zn	Indonesia	2022	(99)
	BRRI Dhan 64	Zn	Bangladesh	2014	(99)
	CR 310, CR 311	Protein	India	2018	(100)
	Zinco Rice MS	Zn	India	2018	(101)
	WB 02, HPBW 01	Fe, Zn	India	2017	(102)
	Pusa Tejas	Protein, Fe	India	2017	(102)
	Pusa Ujala	Protein, Fe, Zn	India	2017	(102)
Wheat	HD 3171	Zn	India	2017	(102)
	Nohely F2018	Zn	Mexico	2018	(99)
	TARNAB-REHBAR	Zn	Pakistan	2023	(99)
	TARNAB-GANDUM-I	Zn	Pakistan	2023	(99)
	Zinc Gahun-1	Zn	Nepal	2020	(99)
	HI 8777	Zn, Fe	India	2018	(102)
	MACS 4028	Protein, Zn and Fe	India	2018	(103)
	PBW 752	Protein	India	2018	(103)
Maize	Vivek QPM 9	lysine and tryptophan	India	2008	(102)
	Pusa HM8 Improved, Pusa HM4	lysine and tryptophan	India	2017	(102)
	Pusa Vivek QPM9 Improved	provitamin-A, lysine and tryptophan	India	2017	(102)
	ZS246A	Vitamin A	Africa	2016	(99)
	ZS500A	Vitamin A	Africa	2019	(99)
	ICTA HB-18ACP + Zn	Zinc	Guatemala	2018	(99)
	Fortaleza 17	Zinc	Guatemala	2020	(99)
	SGBIOH2	Zinc	Colombia	2019	(99)
	Pusa HQPM 5 Improved, Pusa HQPM 7 Improved, IQMH 201	provitamin-A, lysine and tryptophan	India	2020	(2)
Pearl millet	RHB 233, RHB 234	Iron and zinc	India	2019	(103)
	ICSR 14001, ICSH 14002	Iron	India		(104)
	VR 929	Iron	India	2020	(2)
	LCIC MV5	Iron	Nigeria	2023	(99)
	Chakti	Iron	Nigeria	2018	(99)
	CFMV1, CFMV 2	Calcium, iron and zinc	India	2020	(2)
Sorghum	CLMV1	Iron and zinc	India	2020	(2)
	Parbhani Shakti	Zinc	India	2018	(99)
Finger Millet	Pusa Ageti Masoor	Iron	India	2017	(102)
	IPL 220	Iron and zinc	India	2018	(103)
Little Millet	IPL 220	Iron and zinc	India	2018	(103)
*Pulses/Legumes*					
Lentil	Kufri Manik, Kufri Neelkanth	Anthocyanin	India	2020	(2)
	Bhu Sona, Bhu Krishna	Provitamin-A, Anthocyanin	India	2017	(102)
	Rasuwa black	Iron	Nepal	2020	(99)
	Barimasur-4, B-5, B-6	Iron	Bangladesh	2010	(99)
Cowpea	CBC6	Iron	Zimbabwe	2021	(99)
	Pant Lobia-7	Iron	India	2019	(99)
	BRS Araca	Iron	Brazil	2009	(99)
Beans	RWR 2245; RWR 2154; MAC 42; MAC 44; CAB 2; RWV 1129; RWV 3006; RWV 3316; RWV 3317; RWV 2887	Iron and zinc	Rwanda	2014	(99)
*Vegetables*					
Sweet Potato	Delvia	Vitamin A	Zimbabwe	2021	(99)
	Kokota, Chumfwa, Olympia	Vitamin A	Zambia	2014	(99)
	Gerald, Joweria	Vitamin A	Uganda	2013	(99)
Cassava	Slicass 12	Vitamin A	Sierra Leone	2014	(99)
	UMUCASS 44	Vitamin A	Nigeria	2014	(99)
	UMUCASS 52, UMUCASS 53, UMUCASS 54	Vitamin A	Nigeria	2022	(99)
*Fruits*					
Banana	Apantu, Bira, Pelipita, Lai, To’o	Vitamin A	Uganda	(99)	
Mango	Amarpali, Pusa Arunima, Pusa Surya, Pusa Pratibha, Pusa Peetamber, Pusa Lalima, and Pusa Shreshth	Beta-carotene, Vitamin C	India	IARI, India	
	Ataulfo	Beta-carotene, Vitamin C	Mexico	USDA Agricultural Research Service	
Grapes	Pusa Navrang	Antioxidants	India	IARI, India	

Biofortification by breeding is attained when crops have naturally some concentrations of micronutrients, such as iron, zinc and vitamin A, which means when genetic diversity is accessible in usable form. Some examples of biofortified crops include iron-rich beans, zinc-rich rice, selenium-rich rice, wheat and maize, iodine-rich cassava, maize, and sweet potatoes and vitamin A-rich sweet potatoes.

This method is widely accepted as it is safe and does not raise the same safety concerns as genetic engineering. However, traditional breeding can be a slow and labor-intensive process, and it may take many years to develop a crop with improved nutrient content.

## Target crops for biofortification

### Cereals

Rice, wheat and maize are the major calorie supplement for two-thirds of the Indian population thereby ruling the people’s diet in the country. Biofortification of cereals with iron, zinc, protein and provitamin-A content can assist to bring down the issue of hidden hunger in the population who does not have access to diversified food or supplements. Cereals are typically deficient in both protein and vitamin A. Proteins are vital for humans as they build cells, act as enzymes for chemical reactions, regulate hormones, support the immune system, aid in muscle function, and facilitate communication between cells, among other essential roles in maintaining overall health and bodily functions. Dietary protein deficiency can lead to varied effects on body weight and composition. Inadequate protein intake often results in increased food consumption, body weight, and fat mass. Extremely low protein diets cause fatty liver, reduced energy absorption, and persistent decreases in lean mass (107). Vitamin A aids in cell communication, supports reproduction and growth, and acts as an antioxidant to protect cells. Its deficiency can cause problems in the eyes (ophthalmological), skin (dermatological), and immune system (immune impairment) (108).

Polished rice is a poor source of micronutrients; 60–80% of Fe and around 30% of Zn are lost during polishing (109) yet consumers prefer polished rice because of their long storage and their taste preference. Pureline varieties of rice developed by ICAR-IIRR are DRR Dhan 45, DRR Dhan 48 and DRR Dhan 49 having zinc content ranging from 22.6–25.2 ppm. Protein-rich variety CR Dhan 310 has 10.6% protein content in polished rice in comparison to 7.0–8.0% in popular varieties.

Wheat is the second main crop of India after rice. Breeding wheat to improve the quality of the crop has become the recent focus. However, farmers’ acceptance of nutrient-dense cultivars and the introduction of new biofortified varieties into wheat-growing areas is crucial in the fight against hidden hunger (110, 111). Although iron and zinc are abundantly present in the aleurone layer, however, their bioavailability is affected by the presence of phytate (112).

Supplementing with vitamin A presents a problem due to its high cost and need for efficient transportation and storage methods, which are challenging to implement in remote, sparsely populated locations (113). Maize is an ideal crop for biofortifying it with provitamin A due to its natural diversity in carotenoid content, which includes predominant carotenoid components lutein and zeaxanthin, as well as β & α-carotene and β-cryptoxanthin (114). Traditional maize contains less amount of proteins, lysine and tryptophan, over-dependency on this cereal causes diseases such as kwashiorkor and pellagra (115, 116). There are presently several varieties of quality protein maize (QPM) being grown throughout the country having high lysine and tryptophan levels.

### Pulses

Pulses are a major source of protein and other vital nutrients for millions of people, especially in developing countries. Biofortification can effectively address malnutrition by providing more of the key nutrients needed for proper growth, development and overall health. Pulses are not only nutritionally valuable but also environmentally beneficial, as they have the ability to fix nitrogen in the soil, enhancing its fertility. Lentils and beans are particularly promising candidates for enhancing iron and zinc content through conventional breeding methods. These pulses exhibit inherent genetic potential for heightened mineral accumulation. Harnessing this natural richness entails meticulous selection of parental genotypes demonstrating superior mineral concentrations. Through systematic interbreeding over successive generations, novel cultivars can be developed wherein augmented iron and zinc levels are seamlessly integrated while preserving key agronomic attributes. Consequently, these biofortified varieties emerge as pivotal assets for ameliorating micronutrient deficiencies and fostering enhanced nutritional quality within food systems.

### Vegetables and fruits

The consumption of vegetables and fruits is important for a healthy diet, as they are rich in vitamins, minerals, fiber, and other essential nutrients. They offer a diverse, low-calorie, protective and nutrient-dense diet. It has long been understood that eating the recommended amounts of vegetables and fruits has favorable health effects and frequent consumption of a range of these foods has been associated with decreased risk of diseases. The benefits of biofortifying vegetables and fruits include reducing the risk of chronic diseases, increasing economic productivity and promoting overall health and well-being.

### Biofortification through transgenic/biotechnological means

Biotechnology is a field of science that uses living organisms, cells or their components to make useful products or services. It has the potential to solve many of the world’s most pressing problems, such as producing enough food to feed a growing population, developing new treatments for diseases and enhancing the nutritional quality of crops. Biotechnology allows more precise and efficient targeting of specific nutrients than other means.

Nutritional quality in crops can be enhanced either by adding new genes that supply the plants with more vitamins or minerals or by enhancing the expression of already present genes that are involved in nutrient biosynthesis (see Table 7). Transgenic techniques can be used to simultaneously incorporate genes that increase the concentration of micronutrients, their bioavailability and inhibit antinutritional factors (ANFs) in crops that restrict the utilization of nutrients (117). Transgenic approach presents a rational solution to improve the concentration and bioavailability of micronutrients (106, 118) especially when there is a limited genetic base present in different plant varieties (119, 120).

**Table 7:** Genes involved in micronutrient enrichment.

Crops	Genes involved	Micronutrient	References
Wheat	TaVIT1& TaVIT2	Fe and Mn	(121)
	NAM B1, GPC 1 and *PhyA*	Zn and Fe	(122)
	*Ama1*	Tyrosine, lysin, cysteine, methionine	(123)
	*CrtI, CrtB, Bacterial PSY*	Vitamin A	(124)
	*Ferritin TaFer*	Fe	(125, 126)
Rice	OsVIT1, OsVIT2,	Fe	(127)
	OsNAS1, OsNAS2 and OsNAS3	Fe and Zn	(128, 129)
	*AtTC, AtHP*	Vitamin E	(130)
	*THIC, THI1, TH1*	Vitamin B1	(131)
	*AtPDX1.1, AtPDX02*	Vitamin B6	(132)
	*ADCS, AtGTP cyclohydrolase 1*	Vitamin B9	(133)
	*Carotene desaturase, daffodil PSY*	Provitamin A	(134)
	*GmFAD3, ZmC1, chalcone synthase, phenylalanine ammonia* *Lyase*	Flavonoid, linoleic acid	(135)
Maize	*GmFER, aspergillus phytase, aspergillus phy2*	Fe	(117)
	*Zmpsy 1*	Vitamin A	(136)
	*crtI, crtB*	Vitamin A	(124)
	*HGGT*	Vitamin E, tocotrienol	(136)
	*Corynebacterium glutamicum cordapA*	Lysine	(137)
Sorghum	*LPA-1, PMI, PSY-1, CRT-I*	Vitamin A	(138)
Barley	*DHPS*	Lysine, Zn	(117, 139)
	*Phytase, AtZIP*	Fe	(122, 139)
	*Zn transporter gene*	Zn	(117)
	*AtVTE3, AtVTE4*	Tocopherol	(140)
	*HvCs1F*	β-glucans	(140)
*Oilseeds*			
Linseed	*VLCPUFA*	Cholesterol-lowering agents	Newton (141)
	*PSY, crtB*	Carotenoids	(142)
Canola	*PSY, crtB, phytoene desaturase, and lycopene cyclase*	Carotenoids	(142, 143)
	*Aspartokinase (AK) and dihydrodipicolinic acid synthase (DHDPS)*	Lysine	(144)
Mustard	*FAD3*	Linoleic acid	(145)
Soybean	*Phytoene synthase crtB*	β-carotene	(126, 146)
	*FATB1-A and FAD2-1A*	Linoleic acid	(137)
*Vegetables*			
Tomato	*SINCED1*	Vitamin A, pectin and lycopene	(147, 148)
	*Delila, Rosea1, SIANT1*	Anthocyanin	(149, 150)
	*HMT, S3H, SAMT*	Iodine	(151)
Potato	*GBSS*	Starch quality	(152)
	*FPGS, HPPK/DHPS*	Folate	(153)
	*AmA1, tar-1, Boxla, BoxIIa & BoxaIIa-2*	Protein	(3)
	*nptII*	Amylopectin component of starch	(154)
Cauliflower	*Or gene*	β-carotene	(155, 156)
Cassava	*Erwinia crtB phytoene-synthase gene, & Arbidoopsis 1-deoxyxylulose-5-phosphate synthase*	β-carotene	(157, 158)
	*EFA1 gene*	Fe	(159)
	*Ferritin FEA1*	Fe	(157)
	*ASP 1, Zeolin*	Protein	
Sweet potato	*IbMYB1, npt II*	Anthocyanin, carotenoids and antioxidants	(160, 161)
	*Crtl, CrtB, CrtY, LCYe*	ß-Carotene	(126, 162, 163)
*Fruits*			
Apple	*Stilbene synthase*	Antioxidants	(164)
Banana	*PSY2a*	β-carotene	Waltz (165)

Scientists have used biotechnology to develop crops that are high in beta-carotene, a precursor of Vitamin A, iron and zinc which are essential for human health but often lacking in the diet of people in developing countries. One of the examples is the development of vitamin A-rich rice called “golden rice,” which could help to address vitamin A deficiency in developing countries. Additionally, biotechnology can be used to improve the quality of food by increasing its shelf life, enhancing its flavor, reducing its allergenicity and producing food ingredients with health benefits like functional proteins, fibers and lipids. These ingredients can be used to improve the nutritional value of food products, making them more healthful and beneficial for consumers. This can help to make food more accessible and affordable for consumers, particularly in areas where food is scarce or expensive. It is a promising approach to improve the nutritional value of crops, but it is also a controversial issue and further research is needed to fully understand its potential impacts and risks.

### Challenges

There are several challenges that need to be overcome in order to effectively implement biofortification programs:

*Limited availability of biofortified varieties*: Many biofortified crops are still in the development or testing phase and may not yet be widely available for cultivation.

*Limited awareness and understanding of biofortification*: Many people may not be aware of the benefits of biofortified crops or may have misconceptions about their safety or nutritional value.

*Limited distribution and access*: Even if biofortified crops are available, they may not reach the people who need them most, due to factors such as inadequate infrastructure, lack of storage facilities, or high costs.

*Political and regulatory challenges*: The development and distribution of biofortified crops can be hindered by political and regulatory barriers, such as concerns about intellectual property rights, biosafety and trade issues. The development and commercialization of genetically modified (GM) crops, which are a potential tool for biofortification, can be subject to complex and often controversial regulations.

*Agricultural constraints*: Biofortified crops may not always perform as well as non-biofortified varieties under certain growing conditions, such as drought or pests.

*Limited adoption*: Even if biofortified crops are available, farmers may not choose to grow them if they are not familiar with the benefits or if they are not convinced that the crops will be more profitable.

*Consumer acceptance*: Biofortified crops may be perceived as being different or inferior to non-biofortified varieties, which could affect consumer acceptance.

*Funding*: Biofortification programs require ongoing funding in order to support research, development, and implementation efforts.

*Coordination*: Biofortification programs often involve multiple stakeholders, including governments, NGOs, farmers, and the private sector. Ensuring effective coordination among these stakeholders can be challenging.

## Future prospects

The discipline of biofortification has a number of intriguing research areas that hold great potential for the future. It is becoming an increasingly important area of study as the global population continues to hike and the demand for nutrient-rich food is growing. Scientists are working on developing new varieties of crops that are high in essential vitamins and minerals, such as iron, zinc and vitamin A. As research in this field continues to advance, it is likely that we will see an increasing number of nutrient-rich crop varieties that can help to address global malnutrition and improve public health. Improved plant uptake and absorption of crucial nutrients is the subject of another field of biofortification research. This includes the use of fertilizers and other agricultural practices that can increase the availability of nutrients in the soil and enhance the plants’ ability to absorb them.

Overall, the future prospects for biofortification are very promising. Some potential benefits of biofortification include:

*Expanding the range of biofortified crops*: Currently, the main focus of biofortification has been on staple crops such as rice, wheat and maize, but there is potential to biofortify other crops as well, such as fruits, vegetables and legumes.

*Improving the efficiency of biofortification*: Scientists are working on ways to increase the nutrient content of crops using fewer resources, in order to make biofortification more cost-effective and sustainable.

*Improving the distribution and access to biofortified crops*: This may involve developing new storage and transport technologies, as well as working with governments and other organizations to create supportive policies and infrastructure.

*Promoting the awareness and understanding of biofortification*: This could involve educating the public about the benefits of biofortified crops and addressing any concerns or misconceptions about their safety or nutritional value.

*Reducing malnutrition and improving public health*: Biofortification can increase the nutrient value of locally-grown crops, which can help to address deficiencies in essential vitamins and minerals and improve the nutritional status of populations that rely on these crops as a major source of energy and nutrients and contribute to food security by increasing the availability of nutritious foods.

*Supporting sustainable development*: Biofortification can be implemented at a relatively low cost and can be integrated into existing farming practices, making it a sustainable and scalable solution for improving nutrition.

*Improving gender equity*: Women and children are often the most vulnerable to malnutrition, and biofortification can help to reduce gender disparities in access to nutritious foods.

## Conclusion

Biofortification heralds a transformative paradigm in a battle against malnutrition and hidden hunger, which is often not visible to the naked eye, as people may appear well-nourished but still be deficient in essential vitamins and minerals. In some cases, biofortified crops may also have higher yields, which can help to improve food security and increase income for farmers. It can be a cost-effective and sustainable way to improve nutrition, as it relies on using existing agricultural infrastructure and practices. It can help to address dietary deficiencies and improve nutrition in low-income populations, which may not have the same access to nutrient-rich foods as those in higher-income groups. The integration of multi-nutrient biofortification and cutting-edge nano-technology marks a groundbreaking leap.

However, there are several challenges that need to be overcome in order to effectively implement biofortification programs, including limited availability of biofortified varieties, high costs of production, limited awareness and understanding, limited distribution and access, and political and regulatory barriers. Biofortified food crops have the potential to significantly improve the lives and health of millions of underprivileged people in India with careful planning, execution, and implementation while requiring a low investment in research.

## Author contributions

First draft designed by Avnee, reviewed by SS, DC and reviewed and revised by PJ and RR. All authors contributed to the article and approved the submitted version.

## Conflict of interest

The authors declare that the research was conducted in the absence of any commercial or financial relationships that could be construed as a potential conflict of interest.

## Publisher’s note

All claims expressed in this article are solely those of the authors and do not necessarily represent those of their affiliated organizations, or those of the publisher, the editors and the reviewers. Any product that may be evaluated in this article, or claim that may be made by its manufacturer, is not guaranteed or endorsed by the publisher.
